# Perceived Social Support Promotes Nursing Students’ Psychological Wellbeing: Explained With Self-Compassion and Professional Self-Concept

**DOI:** 10.3389/fpsyg.2022.835134

**Published:** 2022-04-05

**Authors:** Lu Zhou, Khunanan Sukpasjaroen, Yuming Wu, Liu Gao, Thitinan Chankoson, Enli Cai

**Affiliations:** ^1^Chakrabongse Bhuvanarth International Institute for Interdisciplinary Studies, Rajamangala University of Technology Tawan-ok, Bang Phra, Thailand; ^2^School of Nursing, Yunnan University of Chinese Medicine, Kunming, China; ^3^Faculty of Business Administration for Society, Srinakharinwirot University, Bangkok, Thailand

**Keywords:** perceived social support, psychological wellbeing, self-compassion, professional self-concept, nursing students

## Abstract

**Background:**

The psychological distress of nursing students is ongoing and getting worse during the coronavirus disease 2019 (COVID-19) outbreak. Numerous calls for future research on exploring the effects of perceived social support would be an effective way to improve nursing students’ mental health. However, the pathway(s) between perceived social support and psychological wellbeing (PWB) remain unknown.

**Objective:**

The aim of this study was to explore how self-compassion and professional self-concept mediate the relationship between perceived social support and PWB to explain the theoretical mechanisms of the relationship.

**Design:**

This study is the analytical cross-sectional research based on online self-reports and completed validated measures of perceived social support, PWB, self-compassion, and professional self-concept.

**Methods:**

The Structural Equation Model (SEM) was used to estimate the mediation effects on the relationship between perceived social support and PWB. To examine the directionality of effects, this study also tested the reverse serial mediation model. Multigroup SEM was used to test gender differences in the mediation model.

**Results:**

The results of an empirical study involving 487 undergraduate nursing students verified an integrative model of social support. In addition, no gender difference was found in these associations. These findings suggest that self-compassion and professional self-concept accounted for the association between perceived social support and PWB, and self-compassion was a significant predictor of professional self-concept.

**Conclusion:**

There is a pathway of self-compassion and professional self-concept through which perceived social support may improve PWB. Also, improving nursing students’ perceived social support, self-compassion and professional self-concept are beneficial for promoting their mental health. It is meaningful for nursing educators to take measures to develop nursing students’ PWB and enhance their professional self-concept.

## Introduction

A workforce shortage and mental health problems of nurses have been major global concerns, particularly during coronavirus disease 2019 (COVID-19) (Lynch et al., 2021). In China, 49.1% of nursing students had an intention for changing majors and 45.4% would not enter into the nursing profession in the future, which may exacerbate nursing workforce shortfalls (Li et al., 2020). Studies had consistently demonstrated that wellbeing plays a vital role in affecting nurses’ decisions to stay in the nursing profession. In addition to stressors that students in many disciplines experience, nursing students experience more pressure, such as death, workplace violence, negative perception of the professional image, and fear of COVID-19 (Li et al., 2020). Poor wellbeing is associated with numerous negative consequences, such as increased risk of suicide and self-harm, limited academic performance, decreased social connectedness, and increased risk of medical errors (Melnyk et al., 2018). Numerous calls for paying attention to social support would be an effective way to improve nursing students’ wellbeing (Li et al., 2020).

Perceived social support can positively predict individual mental health outcomes [i.e., psychological wellbeing (PWB) and subjective wellbeing] (Feeney and Collins, 2015a). However, how perceived social support enhanced mental health is not clear yet. Feeney and Collins (2015a) proposed an integrative model of social support based on the attachment theory, suggesting that there are multiple pathways through which social support promotes wellbeing. According to the theory, the self-evaluation/self-perception pathways map on to self-compassion and professional self-concept, which can promote better wellbeing. This study aims to explore how social support can promote PWB *via* self-evaluation/self-perception pathways, providing empirical evidence for Feeney and Collins’ theoretical model. Also, attention should be paid to how social support enhances PWB *via* nursing students’ self-perceptions, specifically self-compassion and professional self-concept.

### Perceived Social Support

Perceived social support is broadly defined as the perception that one is cared for by others and has a reliable social network that can be turned to in times of need (Taylor, 2011). It was associated with lower levels of negative mental health outcomes (i.e., depression, anxiety, and dysfunctional attitudes) (Feeney and Collins, 2015a). Despite all of this consistent evidence, it remains unknown what mechanism(s) may explain social support benefits to mental health, specifically for nursing students.

For positive mental health outcomes, PWB is in contrast to the view of subjective wellbeing. PWB contains social and personal resources for making progress toward valued goals, and the fulfillment of basic needs for competence, autonomy (AU), and relatedness that promote intrinsic motivation and thriving (Ryff and Keyes, 1995). PWB is viewed as the result of a life well-lived and is an important factor in students successfully adapting to college/university life (Morales-Rodríguez et al., 2020).

Feeney and Collins proposed an integrative model of social support, in which support is a catalyst for promoting better wellbeing through self-perceptions. However, less empirical study has tested this theoretical model or specific pathways. Consequently,

Hypothesis 1 (H1): Perceived social support has both direct and indirect effects on psychological wellbeing among nursing students.

### Self-Compassion

According to this Feeney and Collins’ model, self-perceptions (the second pathway) closely align with the psychological constructs of self-compassion. Self-compassion is showing concern and benevolence to oneself. It involves having self-kindness (SK), a sense of common humanity (CH), and a mindful perspective (Neff, 2008). In fact, self-compassion is associated with positive resilience, self-esteem (Neff, 2008), and greater self-efficacy (Smeets et al., 2014). From the perspective of cognitive neuroscience, self-compassion also engages with a soothing system among the emotion regulatory system that deals with affects such as safety and contentment to have a positive effect on wellbeing (Gilbert, 2010). As such, one is better able to endure negative events and maintain better PWB.

In addition, there are important differences in the modal cultures that exist in nursing and non-medicine that might change the way self-compassion is viewed or experienced. For example, nursing culture tends to be more collectively oriented and traditionally emphasizes compassion (Dev et al., 2020). In addition, nursing students may encounter more challenges in their life than non-medical students (Li et al., 2020).

Social support is associated with increased self-efficacy and self-esteem (Feeney and Collins, 2015b), which leads to more positive self-perceptions. In addition, the quality of social support is presumed to influence internal perceptions of the individual, which in turn reduces psychopathology (Maheux and Price, 2016). That is, higher quality of social support may promote positive self-evaluations.

These studies provide initial evidence that self-compassion could be a mechanism of self-perceptions which in part explains the association between perceived social support and wellbeing. Consequently,

Hypothesis 2 (H2): Self-compassion would independently mediate the relationship between perceived social support and psychological wellbeing among nursing students.

### Professional Self-Concept

Professional self-concept is the subjective experience of nurses, incorporating a self-cognition and a self-evaluation *via* nursing activities, knowledge, and interpersonal relationship around their environment (Angel et al., 2012).

However, the nursing image in China is not always valued by society (Lin et al., 2021). There are negative stereotypes in the perception of nursing (Bennett et al., 2020). In fact, self-concept is a social phenomenon that occurs as a result of interacting with others (Pesut, 2008). The attitude of nursing students is greatly influenced by the image of nursing in the society (Jahromi et al., 2014). Therefore, they are worried about their images as a nurse. This makes them internalize their negative features and have a negative self-concept. According to Feeney, social support will provide recipients with courage, knowledge, resources, or skills to overcome the adverse senses of self-acceptance (SA) to avoid negative impact and maintain a positive attitude.

Limited research has explored the relationship between the professional self-concept of nursing students and the mental health outcomes. One research investigated nursing college students’ self-concept and found that self-concept is negatively related to depression and anxiety (Shang et al., 2017). Furthermore, a more positive self-concept is associated with stronger self-confidence and self-efficacy (Tejpar, 2021). As such, one is better able to deal with challenges and maintain a better adaption to life. Consequently,

Hypothesis 3 (H3): Professional self-concept would independently mediate the relationship between perceived social support and psychological wellbeing among nursing students.

### Self-Compassion: A Predictor of Professional Self-Concept

There is a general consensus that nursing undergraduates face significant challenges (Lin et al., 2021). These forced them to struggle with building a positive professional self-concept to get a stronger clinical competence and a better academic performance (Goliroshan et al., 2021). In fact, self-compassion circumvents comparison with others or is impacted by negative events and focuses on SK/understanding, thus minimizing the distortion of the self-concept (Játiva and Cerezo, 2014). Thus, developing self-compassion could be a good way to help nursing students recover from bad experiences and ambiguous and negative self-concepts.

Self-compassion is also a strong predictor of self-affirmation (Neff, 2008). Self-improvement and self-affirmation from self-compassion encourage positive self-perceptions instead of harsh self-criticism (Neff, 2003).

Although few studies have focused on the relationship between self-compassion and the professional self-concept of nursing students, these studies provide initial evidence that self-compassion would be a predictor of professional self-concept. Consequently,

Hypothesis 4 (H4): Self-compassion and professional self-concept, in sequence, would mediate the association between perceived social support and psychological wellbeing among nursing students.

## Materials and Methods

### Participants and Procedure

Participants were 487 undergraduate nursing students (77.2% females, 74.3% Han Chinese, 81% response rate) recruited from nursing departments of universities in China *via* an online platform of questionnaire data collection. Participants were 56.1% freshmen, 22.3% sophomores, 12.7% juniors, and 8.9% seniors. Participants provided informed consent and then completed an online prescreening questionnaire for the real study and clarified the identity of participants. Fifty-six participants who did not meet the identity verification were excluded from the analyses. The average age was 20 years (SD = 1.65) and ranged from 17 to 25 years. A *post hoc* statistical power analysis was run using the software package G Power (Faul and Erdfelder, 1992). The close-fit and non-close-fit were tested in G Power, and power exceeded 0.99 in both instances. The sample size was between 102 and 142. Therefore, a sample of 487 students has adequate power to detect small effects.

### Measures

#### The Chinese Version of Ryff’s Psychological Wellbeing Scale

The Ryff’s PWB Scale (RPWBS; Ryff and Keyes, 1995) is an 18-item scale on a scale from 1 (strongly disagree) to 6 (strongly agree) used to assess wellbeing by measuring how participants feel about their lives and themselves. Higher scores indicated greater PWB. Confirmatory factor analysis (CFA) was used to evaluate the six-factor structural model of the Chinese version of Ryff’s PWB Scale (C-RPWBS). The C-RPWBS consists of six subscales, namely, positive relations with others (PR), AU, environmental mastery (EM), personal growth (PG), purpose in life (PL), and SA, respectively. The results indicated a fair measurement model fit: χ^2^/df = 2.675, GFI = 0.923, AGFI = 0.899, NFI = 0.877, IFI = 0.914, CFI = 0.933, TLI = 0.884, RMSEA = 0.051, and SRMR = 0.064. Cronbach’s alpha was 0.921.

#### The Chinese Version of the Multidimensional Perceived Social Support Scale

The Multidimensional Perceived Social Support Scale (MSPSS) is composed of 12 items, each with a 7-point Likert-type response option that ranges from 1 (very strongly disagree) to 7 (very strongly agree) (Zimet et al., 1988). The higher the score, the higher the level of perceived social support. The Chinese version of the Multidimensional Perceived Social Support Scale (C-MSPSS) consists of three subscales, namely, significant others (SO), family (FA), and friends (FR), respectively. The results indicated a good measurement model fit: χ^2^/df = 1.847, GFI = 0.921, AGFI = 0.879, NFI = 0.939, IFI = 0.971, CFI = 0.971, TLI = 0.962, RMSEA = 0.069, and SRMR = 0.0428. Cronbach’s alpha was 0.879.

#### The Chinese Version of the Self-Compassion Scale

The Self-Compassion Scale (SCS; Neff, 2008) is a 26-item scale used to assess self-compassion with a 5-point Likert-type score. Response option ranges from 1 (never do that) to 5 (always do that). The negative items were reverse-scored, and the sum across all items was calculated. Higher scores indicated greater self-compassion. This study had translated the scale based on COSMIN (Mokkink et al., 2012). The results indicated that the Chinese version of the Self-Compassion Scale (C-SCS) had a good psychometric property. The C-SCS consists of six subscales, namely, SK, self-judgment (SJ), CH, isolation (I), mindfulness (M), and overidentification (OI), respectively. The results indicated a good measurement model fit: χ^2^/df = 4.532, GFI = 0.931, AGFI = 0.871, NFI = 0.873, IFI = 0.889, CFI = 0.899, TLI = 0.868, RMSEA = 0.074, and SRMR = 0.0436. Cronbach’s alpha was 0.855.

#### The Chinese Version of the Nurses Self-Concept Scale

The Nurses Self-Concept Scale (NSCI; Angel et al., 2012) contained 14 items. Participants indicated their attitude with each item on an 8-point Likert scale ranging from 1 (definitely false) to 8 (definitely true). The Chinese version had been completed a cross-cultural adaptation in 2020. The Chinese version of the Nurses Self-Concept Scale (C-NSCI) consists of four subscales, namely, care (C), knowledge (K), staff relations (SR), and leadership (L). The model fit indexes in this study are as follows: χ^2^/df = 4.989, GFI = 0.799, AGFI = 0.765, NFI = 0.853, IFI = 0.832, CFI = 0.879, TLI = 0.833, RMSEA = 0.094, and SRMR = 0.0836. Cronbach’s alpha was 0.92.

### Analysis

R 2.7.2 was used to calculate sample size *via* G power. IBM SPSS Statistics 23 was used to examine descriptive statistics and correlations. AMOS 23.0 for the Structural Equation Model (SEM) was used to estimate the mediation effects on the relationship between perceived social support and PWB. The Mardia’s coefficients for multivariate kurtosis in the model were >5 (i.e., multivariate kurtosis critical ratio was 49.899) (Byrne, 2010), indicating significant multivariate non-normality in the data. As a result, the Bollen–Stine (B-S) bootstrap *p* procedure was used to adjust model fit and parameter estimates to accommodate the lack of multivariate normality (Bollen and Long, 1993). Multigroup SEM was used to test gender differences in the mediation model.

## Results

### Descriptive Statistics

Descriptive statistics for self-compassion, professional self-concept, perceived social support, and PWB are shown in Table 1. This study found that males’ means and standard deviations were not significantly different from females (*p* > 0.05). However, the level of professional self-concept of nursing students in China was lower than in other countries (Angel et al., 2012).

**TABLE 1:** Descriptive statistics for variables.

Measures	Mean	SD	Variance	Minimum	Maximum	Skewness	Kurtosis	*t*-Value	Female *M* (SD)	Male *M* (SD)	*p*
Self-compassion	3.432	0.604	0.365	1.910	5.000	0.163	0.148	0.859	3.43 ± 0.61	3.50 ± 0.56	0.44
Professional self-concept	6.068	1.182	1.397	1.360	8.000	–0.246	–0.023	0.527	6.07 ± 1.16	6.09 ± 1.39	0.914
Perceived social support	5.179	1.034	1.068	1.170	7.000	–0.673	0.781	1.034	5.16 ± 1.02	5.36 ± 1.12	0.254
Psychological wellbeing	4.391	0.852	0.725	2.390	6.000	–0.135	–0.767	0.802	4.37 ± 0.84	4.60 ± 0.97	0.121

Correlations for all the exogenous and endogenous variables are presented in Table 2. All variables were pairwise related.

**TABLE 2:** Correlations for all variables.

Variable	1	2	3	4
1. Self-compassion	1			
2. Professional self-concept	0.494**	1		
3. Perceived social support	0.407**	0.495**	1	
4. Psychological wellbeing	0.612**	0.616**	0.694**	1

n = 487. **p < 0.01.

### Two-Step Sequential χ^2^ Difference Test Procedure

The two-step modeling approach recommended by Anderson and Gerbing (1988) was employed. The first step is to compare the null model (Mn: no relationship exists among constructs) to the saturated model (Ms: measurement model). The second step is to compare the most theoretical model (Mt) to Ms and Mn models. The results demonstrated that Mt is acceptable, which is presented in Table 3.

**TABLE 3:** Model fit.

Models	χ^2^	χ ^2^/df	GFI	AGFI	IFI	TLI	CFI	RMSEA	SRMR
Ms	966.533	6.620	0.826	0.774	0.897	0.879	0.897	0.108	0.073
Mn	1,892.798	12.453	0.690	0.612	0.781	0.753	0.781	0.154	0.377
Mt	966.533	6.620	0.826	0.774	0.897	0.879	0.897	0.108	0.073

### Path Analysis

**FIGURE 1:**
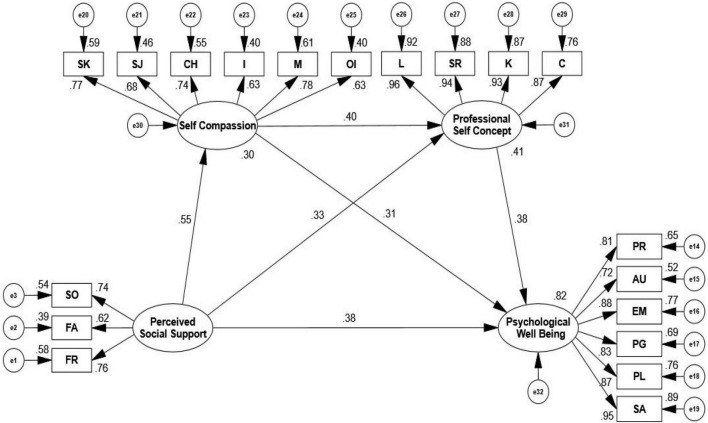
Standardized path coefficient of Structural Equation Model. Self-compassion and professional self-concept in sequence mediated the relationship of perceived social support on psychological wellbeing (PWB). SK, self-kindness; SJ, self-judgment; CH, common humanity; I, isolation; M, mindfulness; OI, overidentification; C, care; K, knowledge; SR, staff relations; L, leadership; SO, significant others; FA, family; FR, friends; PR, positive relations with others; AU, autonomy; EM, environmental mastery; PG, personal growth; PL, purpose in life; SA, self-acceptance.

The hypotheses and the relationships between the variables were significant based on the regression estimates for the accepted structural path model as shown in Table 4. The structural model for the full sample is shown in Figure 1.

**TABLE 4:** Path analysis.

	Unstd. β	SE	t-Value	*p*	Std. β
PSS→SC	0.367	0.039	9.317	***	0.552
PSS→NPSC	0.424	0.074	5.697	***	0.327
SC→NPSC	0.784	0.107	7.345	***	0.402
PSS→PWB	0.337	0.040	8.496	***	0.384
NPSC→PWB	0.259	0.025	10.191	***	0.382
SC→PWB	0.408	0.054	7.613	***	0.309

***p < 0.001.

The B-S bootstrap *p* procedure was used to adjust model fit and parameter estimates to accommodate the lack of multivariate normality. The respecification procedure revealed a considerable model fit increase and produced a theoretically and statistically acceptable final measurement model as reported in Table 5.

**TABLE 5:** Bollen–Stine χ^2^ modify model fit.

GFI	AGFI	NFI	NNFI	IFI	CFI	RMSEA	χ^2^/df	BIC	ECVI
0.977	0.967	0.977	0.994	0.995	0.995	0.024	1.289	460.455	0.567

### Test of Hypotheses

Notably, 95% biased-corrected bootstrapped confidence intervals (CIs) using 5,000 bootstrapped samples with a maximum likelihood model were employed to examine the mediation effects. Table 6 summarizes the results of hypothesis testing. Perceived social support had a positively significant direct effect on PWB (β = 0.337, SE = 0.056, *p* < 0.001). When all mediating variables were simultaneously entered into the equation, the relationship between perceived social support and PWB was still significant (β = 0.334, SE = 0.038, *Z* = 8.789, *p* < 0.001). Thus, Hypothesis 1 was supported. Self-compassion had an independently significant mediation on the relationship between perceived social support and PWB (β = 0.150, SE = 0.027, *^B–C^*95% CI [0.113, 0.203], *^p^*95% CI [0.106, 0.194]). Results using the bootstrap approach showed that the indirect effects of 95% CI did not include 0, and the mediating effect accounted for 22.35% of the total effects and 44.91% of the total indirect effects. In addition, the indirect effect of self-compassion on the relationship between perceived social support and PWB was significantly greater (*p* < 0.05). Therefore, these results were consistent with our Hypothesis 2. Professional self-concept had an independently significant mediation on the relationship between perceived social support and PWB (β = 0.110, SE = 0.028, *^B–C^*95% CI [0.066, 0.158], *^p^*95% CI [0.066, 0.158]), and the mediating effect accounted for 16.39% of the total effects and 32.93% of the total indirect effects, supporting Hypothesis 3. Finally, self-compassion and professional self-concept, in sequence, positively mediated the association between perceived social support and PWB (β = 0.074, SE = 0.019, *^B–C^*95% CI [0.049, 0.114], *^p^*95% CI [0.046, 0.109]), and the mediating effect accounted for 11.03% of the total effects and 22.16% of the total indirect effects. Thus, Hypothesis 4 was supported.

**TABLE 6:** Hypothesis testing.

Effects	Point estimation	Product of Coefficient		Bias-corrected 95% CI			Percentile 95% CI			Ind/Total1	Ind/Total2	Hypothesis	Findings
		SE	*Z*	Lower	Upper	*p*	Lower	Upper	*p*				
**Total effects**												H1	Accepted
Total1	0.671	0.064	10.484	0.571	0.778	0.001	0.573	0.783	0.000	–	–		
**Indirect effects**													
Total2	0.334	0.038	8.789	0.279	0.404	0.000	0.273	0.397	0.000	–	–		
**Direct effects**													
PSSect	0.337	0.056	6.018	0.248	0.433	0.000	0.253	0.440	0.000				
**Mediation effects**													
Ind1: PSS→SC→PWB	0.150	0.027	5.556	0.113	0.203	0.000	0.106	0.194	0.000	22.35%	44.91%	H2	Accepted
Ind2: PSS→PSC→PWB	0.110	0.028	3.929	0.066	0.158	0.000	0.066	0.158	0.000	16.39%	32.93%	H3	Accepted
Ind3: PSS→SC→PSC→PWB	0.074	0.019	3.895	0.049	0.114	0.000	0.046	0.109	0.000	11.03%	22.16%	H4	Accepted
Ind1–Ind2	0.040	0.042	0.952	−0.023	0.116	0.278	−0.032	0.107	0.375	–	–		
Ind1–Ind3	0.075	0.029	2.586	0.032	0.126	0.011	0.025	0.120	0.020	–	–		
Ind2–Ind3	0.035	0.040	0.875	−0.033	0.099	0.394	−0.030	0.100	0.358	–	–		

*5,000 bootstrap samples. SE, standard error of mean.*

**FIGURE 2:**
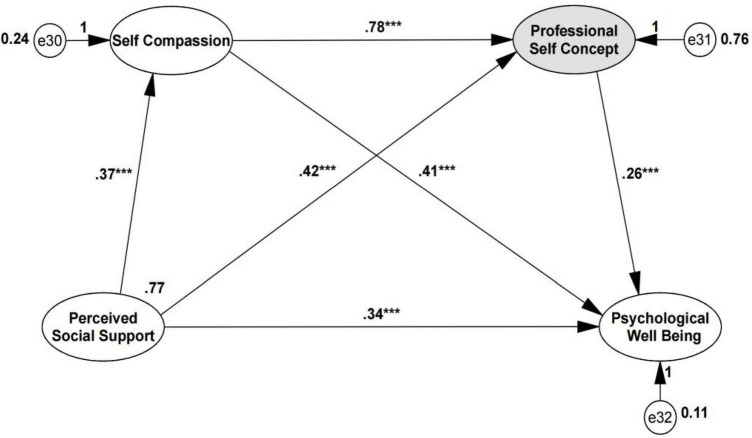
Model of Directionality 1: self-compassion to professional self-concept. After adding the mediators, there is a significant indirect effect of perceived social support on PWB. The coefficients shown above are unstandardized. *Significant at the 0.05 level (two-tailed). **Significant at the 0.01 level (two-tailed). ***Significant at the 0.001 level (two-tailed). The total effects (Ind1 + Ind2 + Ind3 + direct effect) = 0.671. The total indirect effects (Ind1 + Ind2 + Ind3) = 0.334.

**FIGURE 3:**
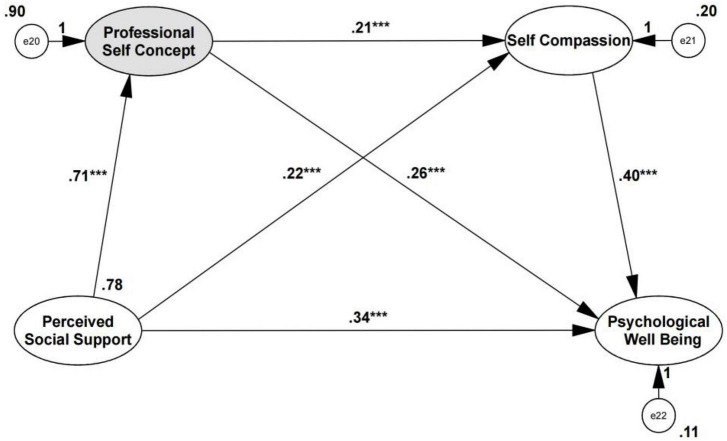
Model of Directionality 2: professional self-concept to self-compassion. After adding the mediators, there is a significant indirect effect of perceived social support on PWB. The coefficients shown above are unstandardized. *Significant at the 0.05 level (two-tailed). **Significant at the 0.01 level (two-tailed). ***Significant at the 0.001 level (two-tailed). The total effects (Ind1 + Ind2 + Ind3 + direct effect) = 0.635. The total indirect effects (Ind1 + Ind2 + Ind3) = 0.224.

Multiple sequential mediation effects were decomposed into direct effects to gain a better understanding of the complex causal relationships between constructs, and the directional test of the serial mediation analyses and the unstandardized path coefficients of SEMs were indicated in Figures 2, 3. The direct effect of self-compassion as the first mediating variable on the second mediating variable of professional self-concept was at the significant level (β = 0.78, *p* < 0.001, SE = 0.107). A review of the direct effects of mediating variables on PWB showed that the effects of self-compassion (β = 0.41, *p* < 0.001, SE = 0.054) and professional self-concept (β = 0.26, *p* < 0.001, SE = 0.025) were significant. These findings indicated that the odds ratio (OR) of PWB reported increased by approximately 41% per unit increase in self-compassion and increased by approximately 26% per unit increase in professional self-concept. In addition, the OR of professional self-concept reported increased by approximately 78% per unit increase in self-compassion. The total effects remained significant (β = 0.671, SE = 0.064, *Z* = 10.484, *p* < 0.001).

For the reverse mediation model (Figure 3), perceived social support was a significant predictor of professional self-concept (β = 0.71, *p* < 0.001, SE = 0.068) and self-compassion (β = 0.22, *p* = 0.009, SE = 0.042). The direct effect of professional self-concept on self-compassion was at the significant level (β = 0.21, *p* < 0.001, SE = 0.029). The direct effects of the mediating variables, professional self-concept (β = 0.26, *p* < 0.001, SE = 0.026) and self-compassion (β = 0.40, *p* < 0.001, SE = 0.053), were both significant and as same as Model of Directionality 1. When perceived social support and all mediating variables were simultaneously entered into the equation, the relationships remained significant. However, the direct effect of professional self-concept on self-compassion was weaker than self-compassion on professional self-concept. In addition, when perceived social support and all mediating variables were simultaneously entered into the equation, the total indirect effects of perceived social support on PWB were also weaker than the Model of Directionality 1. Therefore, self-compassion has a greater direct effect on professional self-concept, and self-compassion was a significant predictor of professional self-concept. Thus, Hypothesis 4 was still supported by these results.

### Invariance of Multigroup Structural Equation Models

Group differences by the moderated mediation model were determined using the multigroup analysis in SEM (Sauer and Dick, 1993). The results showed that the unconstrained model’s and constrained model’s invariance was satisfied in the two groups (ΔCFI < 0.01 and ΔNNFI < 0.05). Models fit and the resulting comparisons of models are presented in Tables 7, 8, respectively.

**TABLE 7:** Models fit.

Models	χ^2^	χ ^2^/df	GFI	IFI	TLI	CFI	RMSEA	SRMR
Model 0	1,372.099	4.604	0.790	0.861	0.839	0.860	0.086	0.105
Model 1	1,394.417	4.523	0.785	0.857	0.843	0.856	0.085	0.114
Model 2	1,395.651	4.516	0.784	0.856	0.843	0.856	0.085	0.113
Model 3	1,401.510	4.488	0.783	0.856	0.844	0.856	0.085	0.115
Model 4	1,535.975	5.128	0.709	0.819	0.816	0.819	0.092	0.115

*Model 0, unconstrained structural model; Model 1, measurement weights’ invariant; Model 2, measurement weights’ and measurement intercepts’ invariant; Model 3, measurement weights’, measurement intercepts’, and structural covariances’ invariant; Model 4, measurement weights’, measurement intercepts’, structural covariances’, and structural residuals’ invariant.*

**TABLE 8:** Model comparisons for multigroup Structural Equation Models.

Comparison	Δχ^2^	Δdf	ΔCFI	ΔNNFI	*p*
Model 0 vs. Model 1	22.318	15	−0.005	–0.005	0.100
Model 0 vs. Model 2	23.552	16	−0.006	–0.005	0.100
Model 0 vs. Model 3	29.411	19	−0.007	–0.005	0.060
Model 0 vs. Model 4	163.876	38	−0.081	0.022	0.000
Model 1 vs. Model 2	1.234	1	−0.001	0.000	0.267
Model 1 vs. Model 3	7.093	4	−0.002	–0.001	0.131
Model 1 vs. Model 4	141.558	23	−0.076	0.029	0.000
Model 2 vs. Model 3	5.859	3	−0.001	0.000	0.119
Model 2 vs. Model 4	140.324	22	−0.075	0.029	0.000
Model 3 vs. Model 4	134.465	19	−0.074	0.030	0.000

*Model 0, unconstrained structural model; Model 1, measurement weights’ invariant; Model 2, measurement weights’ and measurement intercepts’ invariant; Model 3, measurement weights’, measurement intercepts’, and structural covariances’ invariant; Model 4, measurement weights’, measurement intercepts’, structural covariances’, and structural residuals’ invariant.*

## Discussion

This study examined the relationship between perceived social support, self-compassion, professional self-concept, and PWB among nursing students. As hypothesized, perceived social support was associated with better PWB. Perceived social support was also related to a higher degree of self-compassion and a sense of professional self-concept. Both self-compassion and professional self-concept were positively related to PWB. Findings demonstrated that self-compassion and professional self-concept, independently and in sequence, mediated the relationship between perceived social support and PWB. In addition, the effects of the reverse mediation model, with professional self-concept as the first mediator and self-compassion as the second, were less than the hypothetical model, although it was also significant. These findings still held after controlling for gender.

These results demonstrate that self-compassion and professional self-concept may play a role in explaining the relationship between perceived social support and PWB. Professional self-concept was examined as a self-evaluation factor for various mental health outcomes and was shown to explain the effects of close relationships on thriving (Feeney and Collins, 2015a). Similarly, self-compassion is implicated in both interpersonal factors as well as in health and wellbeing (Neff, 2003). Our findings support existing literature highlighting the relevance of these factors to both perceived social support and PWB among nursing students.

Findings from the serial mediation model suggest that nursing students’ self-compassion and professional self-concept may contribute to the relationship between perceived social support and PWB. Professional self-concept mediated the relationship between self-compassion and PWB, while self-compassion has a weaker mediation effect on the relationship between professional self-concept and PWB. These findings suggest that effects between self-compassion and professional self-concept are likely bidirectional. However, based on existing theory and research, a higher degree of self-compassion likely leads to a better sense of professional self-concept. According to Paul Thagard, both self-compassion and professional self-concept are subordinate to self-representing, and there is an interaction mechanism between these two self-related phenomena. That is, there is a positive correlation between nursing students’ self-compassion and professional self-concept (Thagard and Wood, 2015). Neff’s notion model explains the idea behind self-compassion that paradoxically healthy and constructive self-attitudes stem in part from de-emphasizing the separate self. Verplanken and Sui (2019) presented a neurobiological model, which also explains the various functional characteristics of the integrated self, such as emotional and somatosensory connectedness, attention to self-relevant information, and self-positivity (Kuhl et al., 2015; Sui and Humphreys, 2015), meaning that a better self-evaluation product, i.e., self-compassion, increases self-awareness, which leads to a more positive self-concept (Thagard and Wood, 2015). Thus, it is more likely that individuals first develop a positive professional self-concept as a result of a better self-compassion, rather than positive professional self-concept skills improving the self-compassion. It should be noted that the present research has found that self-compassion is a stronger predictor of PWB than both professional self-concept and perceived social support. Future study should sequentially explore the causal and potentially bidirectional relationship between perceived social support, self-compassion, and professional self-concept. Our findings extend the integrative model of social support to the Chinese nursing students’ PWB. Future research should explore trajectories of these various factors across time to better understand their interrelations.

### Limitations

It is also important to acknowledge several methodological limitations to this study. The primary limitation of this study is the use of a cross-sectional sample. While this methodology allowed for a large, diverse sample, it prevents inferences about how variables are causally related as all data were collected at a single time point. It is possible that there were unobserved confounds. Future studies would benefit from the exploration of the causal relationship between perceived social support, self-compassion, professional self-concept, and PWB by asking these research questions in a longitudinal design. The data in this study were obtained from self-reports, which can produce self-report response bias (Paulhus and Vazire, 2007). This study also did not stratify the analyses according to other contextual variables (e.g., universities and grade level), which may affect the standard errors (Webb and Shavelson, 2005).

### Implications

To our knowledge, this is the first study to investigate the relationship between perceived social support, self-compassion, and professional self-concept, as well as to test self-compassion and professional self-concept as factors that explain how perceived social support is related to better PWB. These findings have important implications for guiding practices and policies to enhance Chinese nursing students’ wellbeing, especially during the COVID-19 outbreak. First, our result showed that different sources of perceived social support (e.g., FA and FR) are a significant predictor of mental health; thus, interventions that target to manipulate and enhance social support may be important for nursing students’ mental health. Second, perceived social support can enhance self-evaluations and positive self-cognitive. Building self-compassion and professional self-concept was the effective way to enhance PWB, enabling nursing students to adapt to the stressful life event experiences in their academic and internship and improving their clinical and academic performance (Moreira de Sousa et al., 2018).

Evidence from the present research has several implications for practitioners. First, it is more important to encourage nursing students to obtain social support through different means. Second, universities could set up positive education for undergraduate nursing students, such as compassion cultivation training (CCT) program (Mills and Cooper, 2019), self-care education, and resilience intervention (Mills et al., 2021), promoting self-compassion through nurturing kindness, acceptance, or mindfulness and meditation skills toward the self can aid in the development of a positive view of the self to refer to during negative experiences (Barnard and Curry, 2011). Third, the Bachelor of Nursing program needs to ensure that nursing students develop not only the right values for the nursing profession but also a professional identity based on research, evidence-based practice, and professionalism (Browne et al., 2018). It is also important for educational institutions to be aware of the fact that nursing students’ perceptions of their professional self-concept are constantly changing. It is, therefore, the responsibility of educational institutions to ensure that undergraduate nursing students develop a cohesive professional identity, and it is recommended that training programs in leadership development, critical thinking skills, and a sense of interprofessional collaboration for nursing students be included in the curriculum to promote a positive perception of the professional self-concept. Therefore, increasing nursing students’ awareness of social support and fostering the use of interventions related to self-compassion and professional self-concept may lead to greater PWB and should be integrated into interventions. It is significant for creating a more stable and sustainable nursing workforce for the future.

## Conclusion

Nursing students’ self-compassion and professional self-concept explained the relationship between perceived social support and PWB. Better self-compassion was associated with a more positive professional self-concept. Theoretically, it provides support for Feeney and Collins’ integrative model of social support (Feeney and Collins, 2015a) in cross-culture and cross-population, by suggesting that perceived social support may encourage self-compassion and professional self-concept, which promote PWB. Furthermore, PWB interventions or therapies that incorporate social support may be enhanced by focusing on the aspects of self-evaluations and self-perceptions that aid in the development of self-compassion and professional self-concept among Chinese nursing students.

## Data Availability Statement

The original contributions presented in the study are included in the article/supplementary material, further inquiries can be directed to the corresponding authors.

## Ethics Statement

The studies involving human participants were reviewed and approved by Ethics Committee, Rajamangala University of Technology Tawan-ok. The participants provided their written informed consent to participate in this study.

## Author Contributions

TC and EC: conceptualization. LZ: software and writing—review and editing. LZ and LG: formal analysis. KS, YW, and LG: data curation. EC: supervision. All authors approved the final version of the manuscript.

## Conflict of Interest

The authors declare that the research was conducted in the absence of any commercial or financial relationships that could be construed as a potential conflict of interest.

## Publisher’s Note

All claims expressed in this article are solely those of the authors and do not necessarily represent those of their affiliated organizations, or those of the publisher, the editors and the reviewers. Any product that may be evaluated in this article, or claim that may be made by its manufacturer, is not guaranteed or endorsed by the publisher.
